# Relative risk of second malignant neoplasms highest among young adult cancer patients – a population-based registry study in Finland

**DOI:** 10.2340/1651-226X.2024.34138

**Published:** 2024-06-08

**Authors:** Hanna A. M. Koivisto, Aapeli O. Nevala, Joonas M. Miettinen, Janne M. Pitkäniemi, Nea K. Malila, Sanna M. M. Heikkinen

**Affiliations:** a Faculty of Medicine, University of Helsinki, Helsinki, Finland; b Finnish Cancer Registry, Cancer Society of Finland, Helsinki, Finland; c Faculty of Social Sciences, Tampere University, Tampere, Finland

**Keywords:** Cancer, epidemiology, second malignancy, cohort study

## Abstract

**Background and purpose:**

The objective of this study was to explore the incidence of second malignant neoplasms (SMNs) among adult cancer patients in Finland diagnosed with their first primary cancer (FPC) in 1992–2021.

**Material and methods:**

The study used data from the population-based Finnish Cancer Registry (FCR). Risk estimates were calculated using the standardised incidence ratio (SIR), the ratio of observed second cancers compared to the expected numbers assuming the same cancer incidence as the corresponding sex-age-calendar year -split of the general population.

**Results:**

A total of 573,379 FPCs were diagnosed during 1992–2021. During the follow-up, 60,464 SMNs were diagnosed. Male cancer patients had neither a decreased nor an increased risk (SIR 1.00 [95% CI, 0.99–1.01]) and female patients had an 8% increased risk (SIR 1.08 [95% CI, 1.06–1.09]) of developing any SMN compared to a FPC in the general population. The highest SIR of any SMN was observed in patients aged 20–39 -years at FPC diagnosis, and the SIR decreased by increasing age at diagnosis. Patients with lymphoid and haematopoietic tissue neoplasms, cancers of the mouth and pharynx, endocrine glands, respiratory and intrathoracic organs, skin, and urinary organs had the highest SIRs, while patients with cancers of the male genital organs and the female breast had the lowest SIRs.

**Interpretation:**

Elevated SIRs were observed in cancer patients diagnosed at an early age and for FPCs known to be in large part attributable to lifestyle factors, which highlights the importance of monitoring and encouraging lifestyle changes.

## Introduction

An estimated 19.3 million new cancer cases were diagnosed globally in 2020 [1]. Due to a growing and ageing population, this number is expected to reach 28.4 million by 2040 [1]. In Finland, the number of new cancer cases increased from nearly 20,000 in 1992 to more than 35,000 in 2020, while cancer mortality has decreased in both men and women [2, 3]. There were more than 300,000 cancer patients in Finland in 2020 [3], many of whom have likely received cancer treatment in the form of chemo- or radiotherapy. Increased cancer incidence, in addition to improved survival rates, puts patients at a higher risk of developing second malignant neoplasms (SMN) [1–5].

Several SMN risk factors have been identified. Both chemotherapy and radiotherapy are associated with the development of secondary malignancies [4, 5]. Genetic predisposition, hormonal influence, and lifestyle and environmental factors also play a part [5–7]. Smoking, excess body weight and alcohol consumption are all strongly associated with cancer pathogenesis [6, 7]. Positive associations have also been observed between aforementioned lifestyle factors and the increased risk of several different SMNs [8–12].

In Finland, smoking has been decreasing in men since the 1970s; in women it started to decrease more recently [13, 14]. The proportion of individuals consuming alcohol or having excess body weight has increased since the 1980s [14]. Although modern cancer treatments are increasingly targeted [15], a higher exposure to known lifestyle risk factors, coupled with improving survival rates, is likely to increase the overall incidence of SMNs in Finland [8–14]. While the cost per new cancer patient decreased between 2009 and 2014, the overall costs will increase along with the number of cancer patients [16]. Information about the risk of developing SMNs will thus be of significance when planning future care and preventative measures.

The previous comprehensive registry-based study on the risk of developing SMNs in Finland was based on data from 1953 to 1991 [17]. Many other European studies cover a similar time period or end before 2010 [18–23]. Prominent risk factors, treatments, and survivorship care have changed over time, thus an update is warranted [13–15]. The objective of this population-based study is to explore and describe the incidence of SMNs among adult cancer patients in Finland diagnosed with their first primary cancer (FPC) between 1992 and 2021. The focus will be on the diagnostic age and calendar period of the FPC diagnosis, as well as on the follow-up.

## Material and methods

This study uses data from the nationwide Finnish Cancer Registry (FCR), which maintains information on all cancer cases diagnosed in Finland since 1953. Hospitals, physicians, and laboratories have been obliged to report cancer cases to the FCR since 1961. FCR data includes details on the diagnosis and the tumour, such as the date and method of diagnosis, as well as the topography, morphology and spreading of the tumour. Information on cancer deaths is updated annually from Statistics Finland [24].

The FCR follows the coding rules of the International Agency for Research on Cancer (IARC, 2004) for multiple cancers with modifications [25]. This means multiple primaries in the same organ are mostly excluded, however, with the national Finnish exception of haematological and lymphatic cancers. Both malignant and benign neoplasms of the brain and the central nervous system are registered. Cancers with unclear growth tendencies and in situ- tumours of the bladder and the urinary system are included as well. In addition, certain other benign tumours are also registered, but not reported in routine cancer statistics [3].

**Figure 1:**
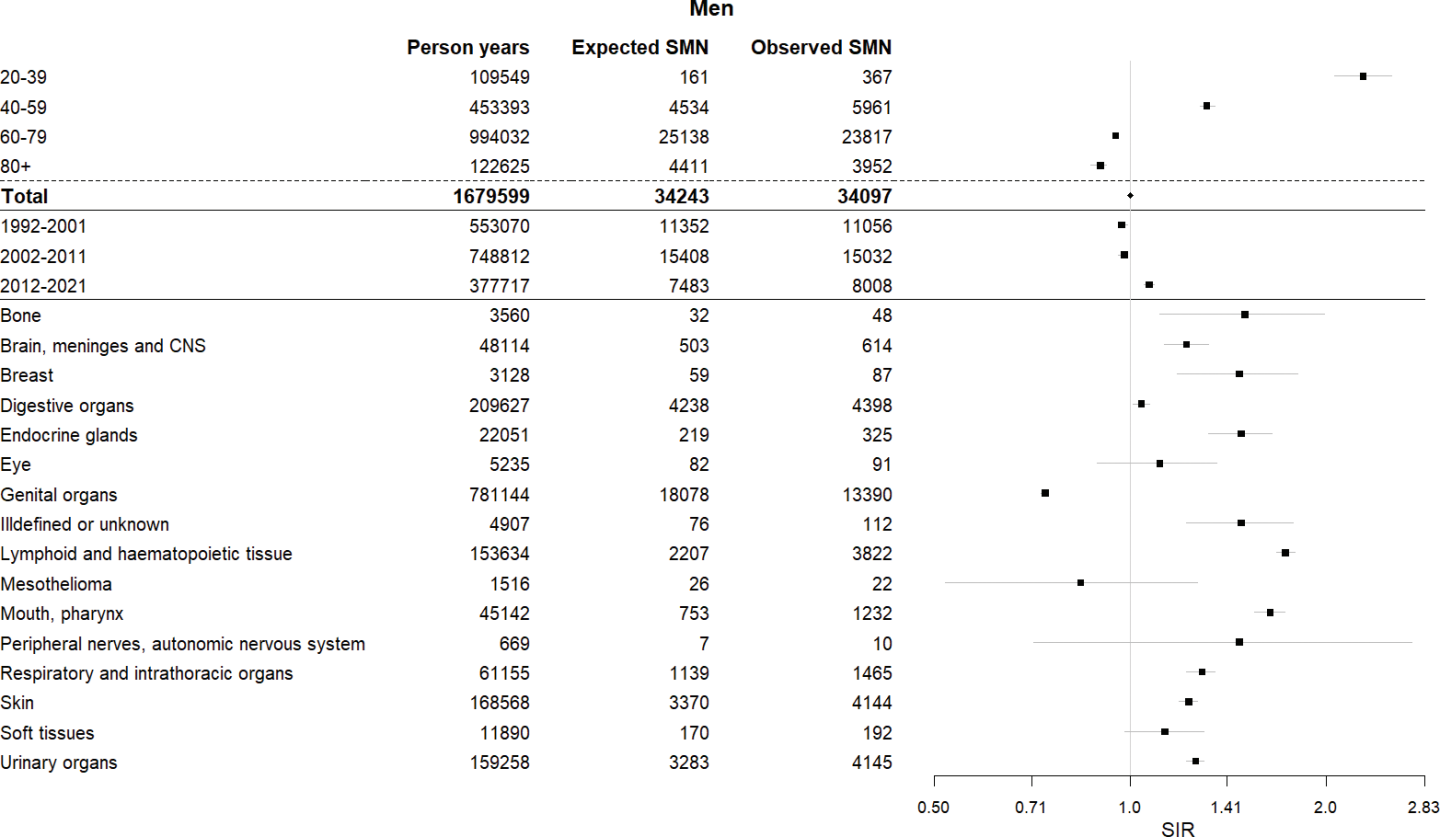
Forest plot showing the standardised incidence ratios (SIR) and confidence intervals (CI) among men for any metachronous second malignant neoplasm (SMN) diagnosed in Finland between 1992 and 2021 by age at first primary cancer (FPC), diagnosis period of FPC and site of FPC.

**Figure 2:**
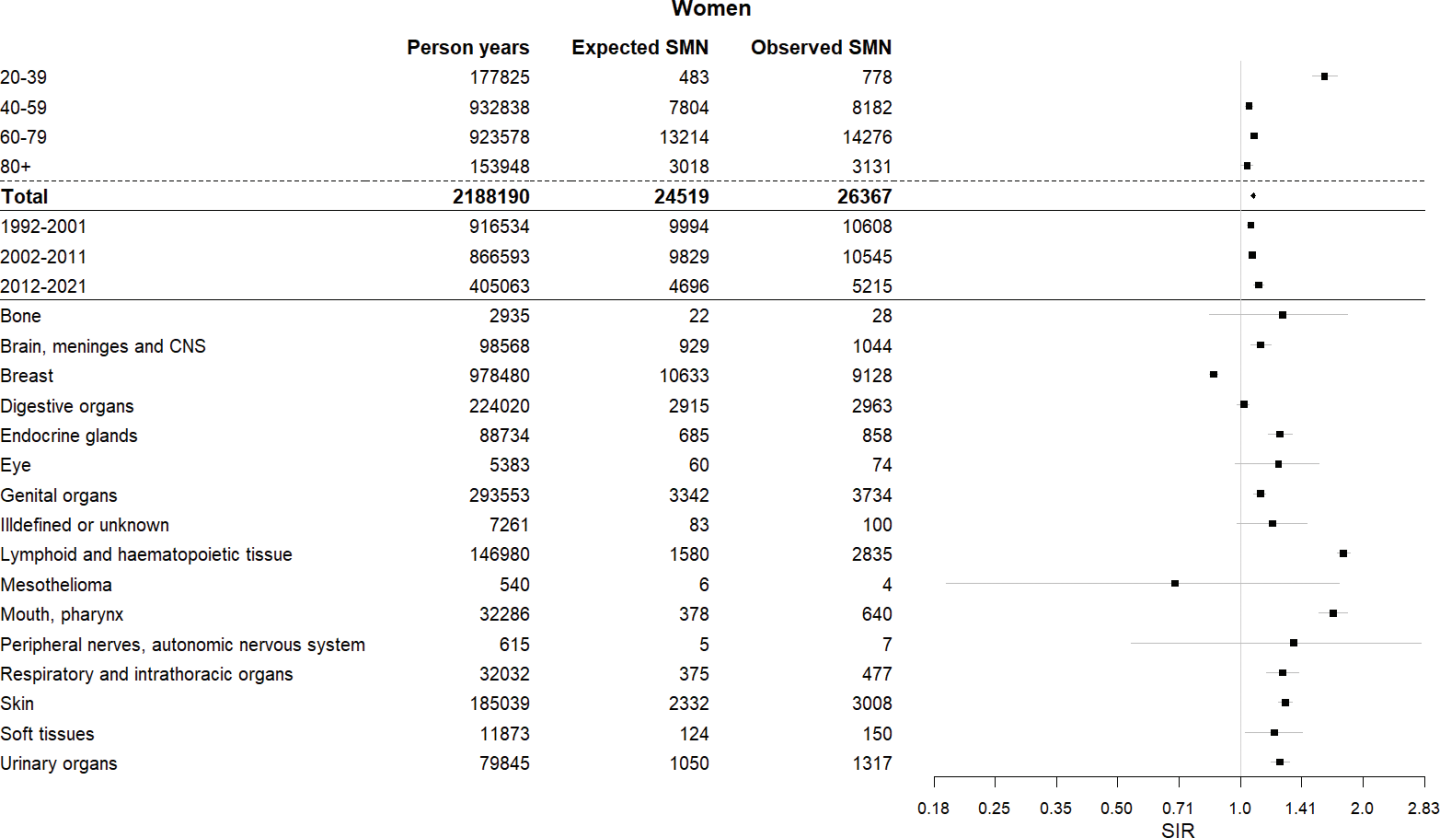
Forest plot showing the standardised incidence ratios (SIR) and confidence intervals (CI) among women for any metachronous second malignant neoplasm (SMN) diagnosed in Finland between 1992 and 2021 by age at first primary cancer (FPC), diagnosis period of FPC, and site of FPC.

In this study, the cancer type classification followed the 10th edition of the World Health Organization’s (WHO) International Classification of Diseases with the following entities included: C00–96, D0.9.0–1, D32–33, D41–43, D45–47, D76 [3]. Cancer primary sites were combined into 16 larger site groups, which will be referred to as primary sites (Table 1, Figures 1 and 2). To avoid synchronous cancers to be included as second malignancies, the follow-up started 6 months after the date of diagnosis of the FPC. FPCs were included until 2021, excluding the last 6 months of the year, and the follow-up ended on 31 December 2021 at the latest.

**Table 1:** Standardised incidence ratios (SIR) for any metachronous second malignant neoplasm (SMN) diagnosed in Finland between 1992-2021 by age at first primary cancer (FPC), diagnosis period of first primary cancer (FPC), and site of first primary cancer (FPC).

Age at FPC, diagnosis period of FPC, and site of FPC	Men						Women					
	No. of FPCs	Obs. SMNs	Exp. SMNs	PYRS	SIR	95% CI	No. of FPCs	Obs. SMNs	Exp. SMNs	PYRS	SIR	95% CI
**Age at FPC**												
20–39	10,312	367	160.79	109,548.57	2.28	2.06-2.52	15,691	778	482.89	177,825.25	1.61	1.50-1.73
40–59	57,308	5,961	4,533.76	453,393.14	1.31	1.28-1.35	89,423	8,182	7,803.87	932,838.44	1.05	1.03-1.07
60–79	177,797	23,817	25,138.00	994,031.93	0.95	0.94-0.96	139,267	14,276	13,214.47	923,578.10	1.08	1.06-1.10
80+	38,933	3,952	4,410.94	122,625.00	0.90	0.87-0.92	44,648	3,131	3,018.13	153,948.41	1.04	1.00-1.07
**Total**	284,350	34,097	34,243.49	1,679,598.65	1.00	0.99-1.01	289,029	26,367	24,519.36	2,188,190.20	1.08	1.06-1.09
**Period of FPC**												
1992–2001	70,462	11,056	11,351.98	553,069.56	0.97	0.96-0.99	79,169	10,608	9,993.89	916,534.50	1.06	1.04-1.08
2002–2011	98,783	15,032	15,408.41	748,812.15	0.98	0.96-0.99	97,168	10,545	9,829.49	866,592.87	1.07	1.05-1.09
2012–2021	115,105	8,008	7,483.10	377,716.95	1.07	1.05-1.09	112,692	5,215	4,695.98	405,062.83	1.11	1.08-1.14
**First primary cancer**												
Bone	470	48	31.96	3,560.39	1.50	1.11-1.99	318	28	22.12	2,935.40	1.27	0.84-1.83
Brain, meninges and CNS	6,909	614	503.04	48,113.91	1.22	1.13-1.32	10,721	1,044	929.20	98,568.46	1.12	1.06-1.19
Breast	467	87	59.24	3,128.46	1.47	1.18-1.81	107,002	9,128	10,633.16	978,479.70	0.86	0.84-0.88
Digestive organs	46,851	4,398	4,238.28	209,626.72	1.04	1.01-1.07	42,944	2,963	2,915.12	224,019.90	1.02	0.98-1.05
Endocrine glands	2,498	325	219.47	22,051.42	1.48	1.32-1.65	7,931	858	685.30	88,734.24	1.25	1.17-1.34
Eye	675	91	82.00	5,235.02	1.11	0.89-1.36	624	74	59.73	5,383.37	1.24	0.97-1.56
Genital organs	111,156	13,390	18,078.24	781,143.62	0.74	0.73-0.75	37,837	3,734	3,342.25	293,552.61	1.12	1.08-1.15
Illdefined or unknown	1,579	112	75.53	4,907.28	1.48	1.22-1.78	2,095	100	83.16	7,261.25	1.20	0.98-1.46
Lymphoid and haematopoietic tissue	25,872	3,822	2,207.22	153,634.50	1.73	1.68-1.79	23,427	2,835	1,579.68	146,980.31	1.79	1.73-1.86
Mesothelioma	987	22	26.30	1,515.54	0.84	0.52-1.27	295	4	5.84	540.00	0.69	0.19-1.75
Mouth, pharynx	7,608	1,232	752.85	45,141.63	1.64	1.55-1.73	4,757	640	378.13	32,285.91	1.69	1.56-1.83
Peripheral nerves, autonomic nervous system	104	10	6.80	669.31	1.47	0.71-2.71	84	7	5.18	615.18	1.35	0.54-2.78
Respiratory and intrathoracic organs	24,262	1,465	1,139.27	61,155.37	1.29	1.22-1.35	10,953	477	374.89	32,032.10	1.27	1.16-1.39
Skin	26,505	4,144	3,369.96	168,567.68	1.23	1.19-1.27	26,575	3,008	2,331.91	185,039.26	1.29	1.24-1.34
Soft tissues	1,826	192	169.97	11,890.26	1.13	0.98-1.30	1,740	150	123.55	11,873.03	1.21	1.03-1,42
Urinary organs	26,581	4,145	3,283.37	159,257.55	1.26	1.22-1.30	11,726	1,317	1,049.96	79,844.99	1.25	1.19-1.32

CI: confidence interval; Exp: expected; FPC: first primary cancer; Obs: observed; PYRS: person-years at risk; SIR: standardised incidence ratio; SMN: second malignant neoplasm.

The follow-up time was considered short when it was up to 5 years, and long if more than 5 years had elapsed since the FPC diagnosis. The diagnostic time of the FPC was divided into three periods, 1992–2001, 2002–2011, and 2012–2021.

We estimated the risk of developing SMNs using standardised incidence ratios (SIR). We split the general population by sex and 5-year intervals by age and calendar period and calculated the incidence rate for a FPC in given strata. This incidence rate multiplied by person-years in the same age-sex-period-strata gave the expected number of cancer cases for the cancer patient cohort. We then calculated the SIR (observed divided by expected number of cases) and confidence intervals (CI) assuming cancer cases are Poisson–distributed. The patient cohort was followed until SMN diagnosis or until the end of the follow-up due to censoring, thus excluding third or a higher number of malignancies. Similarly, for reference rates we followed the general population only until the FPC diagnosis or censoring. Censoring was either due to death unrelated to cancer, emigration from Finland or end of year 2021, whichever was earliest. We chose SIR for estimating the relative risk of SMNs in the cancer prevalent population compared to the general population to evaluate the importance and magnitude of SMNs in the cancer prevalent population, and also to be able to better compare the results with earlier findings in the field, as SIR is frequently used for this purpose [26]. For comparison with other studies in the literature, we also made analyses without restricting the follow-up after the diagnosis of the FPC in the general population, as this seemed to be a commonly used method. Only statistically significant findings are referred to as increased or decreased. All statistical analyses were performed using R version 4.0.2 and R package popEpi 0.4.9.

## Results

There were 573,379 new FPCs (284,350 [50%] in men and 289,029 [50%] in women) diagnosed during 1992–2021 in Finland (Table 1). During the follow-up, 60,464 SMNs were diagnosed (34,096 [56%] in men and 26,368 [44%] in women) (Table 1, Figures 1 and 2), yielding 3,867,789 person-years of follow-up (Table 1, Figures 1 and 2).

The overall risk of developing any SMN after any FPC was neither decreased nor increased among men (SIR, 1.00 [95% CI, 0.99–1.01]) and increased by 8% among women (SIR, 1.08 [1.06–1.09]) (Table 1, Figures 1 and 2). In women, the SIRs were generally increased among patients aged 20–39-, 40–59- and 60–79 -years over all diagnostic periods and follow-up intervals (Tables 2 and 3). In men, the SIRs were increased among those aged 20–39- and 40–59 -years, and generally decreased among those aged 60–79- and 80 and over (Tables 2 and 3). Age-specifically, the SIRs were generally lower in women than men among those aged 20–39- and 40–59 -years, while generally being higher in women than men among those aged 60–79- and 80 and over (Tables 1, 2 and 3, Figures 1 and 2).

**Table 2:** Standardised incidence ratios (SIR) for any metachronous second malignant neoplasm (SMN) diagnosed in Finland between 1992-2021 by age at first primary cancer (FPC) and diagnosis period of first primary cancer (FPC).

Age at FPC	Period of FPC	Men						Women					
		No. of FPCs	Observed SMNs	Expected SMNs	PYRS	SIR	95% CI	No. of FPCs	Observed SMNs	Expected SMNs	PYRS	SIR	95% CI
20–39	1992–2001	2,949	216	103.01	52,234.50	2.10	1.83-2.39	4,993	492	310.77	92,657.25	1.58	1.45-1.73
20–39	2002–2011	3,387	105	41.82	37,380.88	2.51	2.06-3.02	4,851	197	126.46	55,612.97	1.56	1.35-1.79
20–39	2012–2021	3,976	46	15.95	19,933.20	2.88	2.13-3.80	5,847	89	45.65	29,555.04	1.95	1.57-2.38
**20–39**	**1992–2021**	**10,312**	**367**	**160.79**	**109,548.57**	**2.28**	**2.06-2.52**	**15,691**	**778**	**482.89**	**177,825.25**	**1.61**	**1.50-1.73**
40–59	1992–2001	15,416	2,588	2,004.12	171,286.38	1.29	1.24-1.34	27,602	4,195	4,034.11	444,868.30	1.04	1.01-1.07
40–59	2002–2011	22,019	2,435	1,939.74	191,528.22	1.26	1.21-1.31	32,434	2,843	2,775.39	338,172.41	1.02	0.99-1.06
40–59	2012–2021	19,873	938	589.91	90,578.55	1.59	1.49-1.69	29,387	1,144	994.37	149,797.73	1.15	1.09-1.22
**40–59**	**1992–2021**	**57,308**	**5,961**	**4,533.76**	**453,393.14**	**1.31**	**1.28-1.35**	**89,423**	**8,182**	**7,803.87**	**932,838.44**	**1.05**	**1.03-1.07**
60–79	1992–2001	43,207	7,342	8,098.59	299,490.45	0.91	0.89-0.93	35,694	5,106	4,840.89	337,025.92	1.05	1.03-1.08
60–79	2002–2011	60,941	9,702	10,512.23	409,942.10	0.92	0.90-0.94	45,073	5,376	4,957.11	341,375.97	1.08	1.06-1.11
60–79	2012–2021	73,649	6,772	6,527.17	284,599.38	1.04	1.01-1.06	58,500	3,795	3,416.48	245,176.21	1.11	1.08-1.15
**60–79**	**1992–2021**	**177,797**	**23,816**	**25,138.00**	**994,031.93**	**0.95**	**0.94-0.96**	**139,267**	**14,277**	**13,214.47**	**923,578.10**	**1.08**	**1.06-1.10**
80+	1992–2001	8,890	910	1,146.26	30,058.23	0.79	0.74-0.85	10,880	815	808.13	41,983.04	1.01	0.94-1.08
80+	2002–2011	12,436	1,419	1,555.51	43,297.88	0.91	0.87-0.96	14,810	1,143	1,092.50	55,384.48	1.05	0.99-1.11
80+	2012–2021	17,607	1,623	1,709.18	49,268.90	0.95	0.90-1.00	18,958	1,173	1,117.51	56,580.88	1.05	0.99-1.11
**80+**	**1992–2021**	**38,933**	**3,952**	**4,410.94**	**122,625.00**	**0.90**	**0.87-0.92**	**44,648**	**3,131**	**3,018.13**	**153,948.41**	**1.04**	**1.00-1.07**
**All ages**	**1992–2021**	**284,350**	**34,096**	**34,243.49**	**1,679,598.65**	**1.00**	**0.99-1.01**	**289,029**	**26,368**	**24,519.36**	**2,188,190.20**	**1.08**	**1.06-1.09**

CI: confidence interval; Exp: expected; FPC: first primary cancer; Obs: observed; PYRS: person-years at risk; SIR: standardised incidence ratio; SMN: second malignant neoplasm.

Results for all three diagnostic periods combined bolded.

**Table 3:** Standardised incidence ratios (SIR) for any metachronous second malignant neoplasm (SMN) diagnosed in Finland between 1992-2021 by follow-up interval and age at first primary cancer (FPC).

Follow-up interval	Age at FPC	Men					Women				
		Observed SMNs	Expected SMNs	PYRS	SIR	95% CI	Observed SMNs	Expected SMNs	PYRS	SIR	95% CI
0.5–5 years	20–39	80	24.72	37,884.73	3.24	2.58-4.00	175	75.25	59,420.39	2.33	2.00-2.69
> 5 years	20–39	287	136.07	71,663.84	2.11	1.87-2.36	603	407.64	118,404.86	1.48	1.36-1.60
All	20–39	367	160.79	109,548.57	2.28	2.06-2.52	778	482.89	177,825.25	1.61	1.50-1.73
0.5–5 years	40–59	1,606	1,064.65	185,783.99	1.51	1.44-1.58	2107	1,932.10	329,456.25	1.09	1.04-1.14
> 5 years	40–59	4,355	3,469.12	267,609.15	1.26	1.22-1.29	6075	5,871.76	603,382.19	1.03	1.01-1.06
All	40–59	5,961	4,533.76	453,393.14	1.31	1.28-1.35	8182	7,803.87	932,838.44	1.05	1.03-1.07
0.5–5 years	60–79	11,258	11,818.31	528,047.72	0.95	0.94-0.97	5868	5,569.95	439,822.52	1.05	1.03-1.08
> 5 years	60–79	12,558	13,319.69	465,984.22	0.94	0.93-0.96	8409	7,644.52	483,755.58	1.10	1.08-1.12
All	60–79	23,816	25,138.00	994,031.93	0.95	0.94-0.96	14277	13,214.47	923,578.10	1.08	1.06-1.10
0.5–5 years	80+	2,949	3,312.96	92,387.55	0.89	0.86-0.92	2169	2,101.83	109,319.59	1.03	0.99-1.08
> 5 years	80+	1,003	1,097.98	30,237.46	0.91	0.86-0.97	962	916.30	44,628.81	1.05	0.98-1.12
All	80+	3,952	4,410.94	122,625.00	0.90	0.87-0.92	3131	3,018.13	153,948.41	1.04	1.00-1.07
0.5–5 years	All	15,893	16,220.63	844,104.99	0.98	0.96-1.00	10319	9,679.14	938,018.75	1.07	1.05-1.09
> 5 years	All	18,203	18,022.86	835,494.67	1.01	1.00-1.02	16049	14,840.22	1,250,171.45	1.08	1.06-1.10
All	All	34,096	34,243.49	1,679,598.65	1.00	0.99-1.01	26368	24,519.36	2,188,190.20	1.08	1.06-1.09

CI: confidence interval; Exp: expected; FPC: first primary cancer; Obs: observed; PYRS: person-years at risk; SIR: standardised incidence ratio; SMN: second malignant neoplasm.

When stratifying the analyses into short (0.5–5 years) and long (over 5 years) follow-up intervals, SIRs among those aged 20–39- and 40–59 -years tended to decrease with a longer follow-up time (Table 3).

SIRs decreased by diagnostic age at FPC among men and women, in men from SIR 2.28 (2.06–2.52) among those aged 20–39 -years to 0.90 (0.87–0.92) among those aged 80 and over, and in women from SIR 1.61 (1.50–1.73) to 1.04 (1.00–1.07), respectively (Table 1, Figures 1 and 2). The decrease was seen over both short and long follow-up intervals (Table 3) and over all studied calendar periods (Table 2).

When stratifying the analyses by the FPC site groups, many of the SIRs for any SMN were increased (Table 1, Figures 1 and 2). The highest SIRs were observed for FPCs of the lymphoid and haematopoietic tissue (SIR 1.73 [1.68–1.79] in men and 1.79 [1.73–1.86] in women), the mouth and pharynx (SIR 1.64 [1.55–1.73] and SIR 1.69 [1.56–1.83]), the endocrine glands (SIR 1.48 [1.32–1.65] and SIR 1.25 [1.17–1.34]), the respiratory and intrathoracic organs (SIR 1.29 [1.22–1.35] and SIR 1.27 [1.16–1.39]), the skin (SIR 1.23 [1.19–1.27] and SIR 1.29 [1.24–1.34]), and the urinary organs (SIR 1.26 [1.22–1.30] and SIR 1.25 [1.19–1.32]), in addition to the breast in males (SIR 1.47 [1.18–1.81]) (Table 1, Figures 1 and 2). However, the largest number of SMNs was observed in cancer cases of the male genital organs and the female breast even if the respective SIRs were low (SIR 0.74 [0.73–0.75] and SIR 0.86 [0.84–0.88]) (Table 1, Figures 1 and 2).

In men, the SIR decreased by age at first diagnosis for FPCs of the digestive organs, lymphoid and haematopoietic tissue, the male genital organs, and the respiratory and intrathoracic organs (Supplement 1). It is of note that the SIR for the male genital organs changed from increased in men aged 20–39-years (SIR 2.07 [1.60–2.64]) to decreased in 40–59-years and older (SIR 0.88 [0.84–0.93], 0.73 [0.71–0.74] and 0.72 [0.68–0.75], respectively). In women, the SIR decreased by age at first diagnosis for FPCs of the breast, the digestive organs, lymphoid and haematopoietic tissue, the skin, the urinary organs, and the brain, meninges and central nervous system (Supplement 1).

In the supplementary analysis, where the follow-up was extended beyond the FPC in the general population, we observed a 19% increased risk of developing any SMN among men (SIR 1.19 [1.18–1.20]) and 22% among women (SIR 1.22 [1.21–1.24]) (Supplement 2).

## Discussion

We found male cancer patients to have neither an increased nor a decreased SMN risk (SIR 1.00) and females cancer patients to have an 8% increased risk (SIR 1.08) compared to a FPC in the general population. The highest risk of any SMN was in those aged 20–39-years at FPC diagnosis, and the risk decreased by increasing age at FPC diagnosis. The risk of any SMN was highest for patients with a FPC of the lymphoid and haematopoietic tissue, mouth and pharynx, endocrine glands, respiratory and intrathoracic organs, skin, and urinary organs, in addition to the male breast. By contrast, the risk of any SMN was lowest for cancers of the male genital organs and the female breast, with reduced risk estimates from ages 40 and over.

We found higher overall SIRs in women than in men, matching earlier findings from Finland (1.00 in men and 1.25 in women) [17]. Previous studies in Sweden (SIR 1.3 vs 1.6), Austria (SIR 0.90 vs 1.00), Italy (SIR 0.78 vs 0.96), and the US (SIR 1.01 vs 1.10) have yielded similar results [19–21, 27]. Studies conducted in Switzerland (SIR 1.18 vs 1.20) and France (SIR 1.38 vs 1.32), however, found no statistically significant difference in SIRs between the sexes due to overlapping confidence intervals [18, 22].

Explanations could include women being more susceptible to SMNs after radiotherapy and exposed to radiation at younger ages than men due to the incidence of breast and thyroid cancer [5]. Furthermore, hormonal drugs such as tamoxifen used to treat breast cancer increase the risk of endometrial cancer [6]. In contrast, lifestyle and environmental factors associated with the development of SMNs are more commonly linked to men than women. Indeed, SMNs after FPCs associated with smoking, alcohol consumption and obesity skewed the results heavily towards men (Table 1, Figures 1 and 2) [6–14].

The difference in overall SIRs between men and women is also likely caused by the SIR for the male genital organs being lower than that for female breast cancer, as these two groups constituted the highest number of FPCs overall (Table 1). Both of these SIRs being decreased likely also explains the observed disparity between the relatively low overall SIR and high site-specific SIRs (Table 1, Figures 1 and 2). Using the IARC’s rules for reporting multiple primaries excludes cancers of the same histological group arising in a pair of organs, thereby possibly decreasing the number of second breast tumours and reducing the SIR [25]. The low SIR after the male genital organs is likely due to prostate cancer making up the majority of cases in men aged 40 and over [2]. Increasing age is one of the most important risk factors associated with prostate cancer, and as we reported the risk by the specific first primary site, these individuals would have no previous cancer history [2, 28]. Moreover, prostate cancer is commonly diagnosed in its latent phase, making some conditions less aggressive and the treatment conservative [28–30]. This could imply less common treatment related risk factors in many of the patients, and therefore a lower likelihood of developing an SMN. It is of note that studies from the US and Austria have presented separate SIRs both including and excluding prostate cancer, with the SIR excluding prostate cancer increasing from 1.01 to 1.11 in the US and 0.90 to 1.10 in Austria. This suggests that male genital organs could have affected our overall results as well [20, 27].

All FPC sites with the highest SIRs for SMNs have been previously connected to smoking, alcohol consumption and excess body weight, with smoking being especially prevalent (Table 1, Figures 1 and 2) [6–12]. Risk factors for FPC sites are also risk factors for SMNs, which relates to the risk of cancer due to habits such as smoking and other long-term lifestyle-related factors with long-lasting effects. Certain treatments have been associated with an increased risk of SMNs as well, with radiotherapy emphasised in long-term outcomes and the risk of solid malignancy, and chemotherapy emphasised in short-term outcomes, as well as the risk of lymphoid and haematological malignancies specifically [4–6].

The primary sites with the highest SIRs were mostly in line with earlier findings, as was the low SIR after a FPC of the male genital organs [17–23, 27]. The fluctuations in site-specific risk estimations between studies were most likely affected by how sites were grouped together, where high SIR sites combined with low SIR sites could lead to less emphasised results.

In the case of breast cancer, our results differed from many others, with the risk of SMNs usually being increased instead of decreased [17, 19, 21–23, 26]. A previous study including patients from multiple Nordic population-based registries also reported an increased risk of SMNs after breast cancer (SIR 1.15 [95% CI, 1.14–1.17]) [31]. Only an Austrian study found the risk of SMNs after breast cancer to be decreased, as did we [20]. Similarly to us, Austria followed the IARC rules excluding bilateral breast cancers (except if different histological group), but so did most previous studies. Thus, this alone does not explain the differences in results [25].

Similarly to the earlier Finnish findings, the SIR was highest in patients aged 20–39 -years and the risk declined by age [17]. This is known to be at least partly caused by an increased susceptibility to radiotherapy [5]. Furthermore, familial aggregation is more often associated with early onset cancers [32]. The SIR was also found to decrease by age at FPC diagnosis in Switzerland, Austria, Italy and France [18–22].

In our case, the primary sites with the highest SIRs for SMNs among 20–39 -years were lymphoid and haematopoietic tissue in both sexes, in addition to the endocrine glands, digestive organs, male genital organs, and mouth and pharynx in men, as well as skin, soft tissues, and the brain, meninges and central nervous system in women, which are common cancers in adolescents and young adults (Supplement 1) [33, 34]. The distribution of different cancer types, grouped together as one FPC site, varies between age groups, like that of the male genital organs. Among men aged 40 and over, prostate cancer makes up the majority of cases, decreasing the SIR, whereas in 20–39 -year-olds testicle cancer is the major primary site, increasing the SIR [2].

Direct comparisons between studies are difficult to make due to methodological differences. For example, in the earlier Finnish study the follow-up of the general population did not end at FPC diagnosis and therefore resulted in lower expected numbers with higher SIRs than the present analyses [17]. This method seemed to be the more commonly used alternative, as we could only find mentions of restricting follow-up to the general population in a Danish study from 2012 [18–23, 27]. This does, however, not account for the lower SIRs in Austria’s and Italy’s results [20, 21]. Further complicating comparisons, we evaluated the risk of any SMN by FPC site, whereas, for example, the earlier Finnish study reported SIRs for specific SMNs after any FPC [17]. Many previously mentioned studies calculated risks for both selected specific FPCs and specific SMNs [18–23, 27]. Denmark used hazard ratios for comparing risk between cancer patients and a matched sample of the general population [23]. More recently, cumulative incidence has been used for risk estimations as well, comparing the risk within a cohort of cancer patients. In this case, we preferred using SIR, because we wanted to compare the risk between cancer patients and the general population instead [35].

Although overall second malignancy risk estimations may vary, the common trends point toward similar results. Both primary and secondary cancer sites associated with smoking, alcohol use and obesity generally score the highest risk estimates, while cancers of the male genitals, specifically prostate cancer, seem to result in some of the lowest risk estimates.

The present study used data from the FCR, which is legally based and registers all incident cancers since 1953 in Finland. The FCR follows WHO guidelines for coding, including those for multiple primary malignancies. The national coverage of cancer cases is high and the losses in follow-up practically none. The overall completeness of the cancer registry is 96% for solid tumours and 86% for non-solid tumours. Yet, while it generally provides accurate and near complete national cancer data on solid malignancies, it has some weaknesses in the case of tumours not histologically verified, such as haematological malignancies [24]. Death certificates with cancer mentioned, information on deaths and migration are transferred to the registry regularly, which increases the coverage and completes the follow-up [24]. The high coverage and valid coding combined with the homogeneity of the Finnish population [36], make results generalisable to predominantly Caucasian populations and in terms of lifestyle and other environmental factors, to areas with a very high human development index [37].

The median latency for case verification is 18 months among European cancer registries, which stands true for the FCR as well. There is also a 3–6-month delay before data is published [24]. Details such as treatment information are of low accuracy compared to the clinical records [38]. Our study also lacks information on risk factors, such as smoking history, alcohol consumption or BMI.

Among adult cancer patients in Finland, the overall risk of developing a SMN was slightly increased among women and neither increased nor decreased among men compared to developing a FPC in the general population. However, the risk was materially increased in patients diagnosed at young ages and for primary cancers known to be in large part attributable to lifestyle factors such as smoking, alcohol consumption, and obesity. These risk groups highlight the importance of continued monitoring, choice of treatment and encouraging of lifestyle changes among long-term cancer patients.

## Author contributions

NM, JP, SH, and JM developed the concept and study design. HK drafted the first version of the manuscript with support from SH and NM. AN and JM conducted the statistical analyses with supervision from JP. All authors contributed to interpretation of the results, critically reviewed the manuscript, and approved the final manuscript before submission.

## Supplementary Material

Relative risk of second malignant neoplasms highest among young adult cancer patients – a population-based registry study in Finland

Relative risk of second malignant neoplasms highest among young adult cancer patients – a population-based registry study in Finland

## Data Availability

The data that support the findings of this study are available from the corresponding author upon reasonable request.
